# A Genome-Wide Test of the Differential Susceptibility Hypothesis Reveals a Genetic Predictor of Differential Response to Psychological Treatments for Child Anxiety Disorders

**DOI:** 10.1159/000444023

**Published:** 2016-04-05

**Authors:** Robert Keers, Jonathan R.I. Coleman, Kathryn J. Lester, Susanna Roberts, Gerome Breen, Mikael Thastum, Susan Bögels, Silvia Schneider, Einar Heiervang, Richard Meiser-Stedman, Maaike Nauta, Cathy Creswell, Kerstin Thirlwall, Ronald M. Rapee, Jennifer L. Hudson, Cathryn Lewis, Robert Plomin, Thalia C. Eley

**Affiliations:** ^a^Department of Biological and Experimental Psychology, School of Biological and Chemical Sciences, Queen Mary University of London, UK; ^b^MRC Social, Genetic and Developmental Psychiatry (SGDP) Centre, Institute of Psychiatry, Psychology and Neuroscience, King's College London, London, UK; ^c^University of Sussex, Brighton, UK; ^d^University of East Anglia, Norwich, UK; ^e^School of Psychology and Clinical Language Sciences, University of Reading, Reading, UK; ^f^Department of Psychology, University of Aarhus, Aarhus, Denmark; ^g^Research Institute of Child Development and Education, University of Amsterdam, Amsterdam, The Netherlands; ^h^University Medical Center Groningen, University of Groningen, Groningen, The Netherlands; ^i^Ruhr-Universität Bochum, Bochum, Germany; ^j^Institute of Clinical Medicine, University of Oslo, Oslo, Norway; ^k^Anxiety Research Network, Haukeland University Hospital, Bergen, Norway; ^l^Centre for Emotional Health, Department of Psychology, Macquarie University, Sydney, N.S.W., Australia

**Keywords:** Differential response, Child, Anxiety disorders

## Abstract

**Background:**

The differential susceptibly hypothesis suggests that certain genetic variants moderate the effects of both negative and positive environments on mental health and may therefore be important predictors of response to psychological treatments. Nevertheless, the identification of such variants has so far been limited to preselected candidate genes. In this study we extended the differential susceptibility hypothesis from a candidate gene to a genome-wide approach to test whether a polygenic score of environmental sensitivity predicted response to cognitive behavioural therapy (CBT) in children with anxiety disorders.

**Methods:**

We identified variants associated with environmental sensitivity using a novel method in which within-pair variability in emotional problems in 1,026 monozygotic twin pairs was examined as a function of the pairs' genotype. We created a polygenic score of environmental sensitivity based on the whole-genome findings and tested the score as a moderator of parenting on emotional problems in 1,406 children and response to individual, group and brief parent-led CBT in 973 children with anxiety disorders.

**Results:**

The polygenic score significantly moderated the effects of parenting on emotional problems and the effects of treatment. Individuals with a high score responded significantly better to individual CBT than group CBT or brief parent-led CBT (remission rates: 70.9, 55.5 and 41.6%, respectively).

**Conclusions:**

Pending successful replication, our results should be considered exploratory. Nevertheless, if replicated, they suggest that individuals with the greatest environmental sensitivity may be more likely to develop emotional problems in adverse environments but also benefit more from the most intensive types of treatment.

## Introduction

Anxiety disorders are among the most prevalent of all mental illnesses and some of the earliest to emerge, with the vast majority of adult cases beginning in childhood or adolescence [1]. While there is a substantial evidence base for the efficacy of psychological treatments for anxiety in children, response to treatment varies substantially between patients [2]. This means that identifying an effective treatment can be a long and costly process of trial and error that may both delay recovery and have a negative effect on long-term outcomes. Genetic predictors of treatment response may allow clinicians to select the most effective treatment for a given individual at the outset, enhancing outcomes and accelerating recovery times [3]. Such predictors could also offer valuable insights into the mechanisms underlying response to psychological treatments [4].

The differential susceptibility hypothesis suggests that genetic factors moderate the effects of both negative and positive environments on mental health - for better and for worse [5]. In line with this hypothesis, individuals with 1 or 2 copies of the short allele of the 5-HTTLPR have been shown to be at greater risk for mood disorders following adversity than individuals homozygous for the long allele [6]. However, these same individuals also benefit more from positive environmental influences such as supportive parenting [7], positive life events [8] and social support [9]. Importantly, these associations have also been shown to extend to moderation of the positive effects of various interventions including psychosocial training on depression [10], high-quality foster care on disturbances of attachment [11] and externalizing behaviour [12], and the efficacy of cognitive behavioural therapy (CBT) in children with anxiety disorders [13]. In addition to findings from the 5-HTTLPR, differential susceptibility has been reported for a small number of further markers [14], with results from intervention studies showing particular promise [15]. Nevertheless, findings have failed to replicate, even in high-quality studies, with very similar methodologies [16,17]. While the causes of non-replication are unclear, one explanation is that environmental responsivity is a complex, polygenic trait, which is the result of multiple genetic variants of small effect rather than a few select candidate genes.

This suggests that gene-environment interaction should move from a candidate gene to genome-wide approach taking to account the aggregate effects of multiple variants [18].

Polygenic scoring allows the effects of multiple variants to be summarized in a single score. Specifically, alleles associated with a trait in a discovery sample at a given p value threshold are selected in an independent validation sample, and a score (the sum of these alleles weighted by their effect size) is created for each individual [19]. Using this approach, a recent study reported that a polygenic score calculated using the results of a large case-control study of major depression moderated the effects of childhood maltreatment on depression in a further sample, with the interaction explaining a further 0.5% of the variance [20]. This approach to whole-genome gene-environment interaction relies on the assumption that genetic variants have a main effect on outcome. This means that while this method may be suitable for detecting variants implicated in diathesis-stress interactions, it may not detect those involved in differential susceptibility, which are proposed to have no main effects on the phenotype [5]. One means of targeting these variants is to explore genetic effects on intra-pair variability in outcomes in monozygotic (MZ) twin pairs. As they are genetically identical and share the same environment, discordance within MZ twin pairs on a measured outcome is considered to be the result of non-shared environmental effects. Genetic variants that increase sensitivity to the environment should therefore also increase discordance within MZ twin-pairs, as they render each member of the pair more responsive to non-shared environmental influences [21]. While this method has been previously used in a genome-wide study of metabolism [22], it is yet to be applied to analyses of mental health outcomes. Moreover, this approach is yet to incorporate polygenic scoring to consider of the aggregate effects of variants associated with environmental sensitivity.

In this study we aimed, for the first time, to test the differential susceptibility hypothesis using a genome-wide approach. First, we examined associations between genetic variants and intra-pair variability in emotional problems in MZ twins using genome-wide data. Next, in order to validate these findings, we calculated a polygenic score of sensitivity to the environment and tested whether this score moderated the effects of positive and negative parenting on emotional problems in a further sample of children. Finally, to test whether these same variants moderated response to psychological treatment, we tested the polygenic environmental sensitivity score as a predictor of treatment response in a sample of children and adolescents with anxiety disorders treated with individual CBT, group CBT or brief parent-led CBT.

In addition to examining the effect of the polygenic environmental sensitivity score on overall treatment response, we also explored whether the polygenic score predicted a differential response to the different types of treatment received. The effect of environmental sensitivity on response to psychological treatments with differing intensities remains unknown. It has been suggested that those with a low sensitivity to the environment may require a more intensive type of treatment to achieve the same results as those who are highly sensitive. In this case, individuals with a low sensitivity would respond better to individual CBT than brief parent-led CBT. Conversely, it has also been argued that individuals with a high sensitivity to the environment may benefit the most from more intensive forms of treatment. In this case individuals with a high sensitivity would respond more favourably to individual CBT compared to lower-intensity treatments such as brief parent-led CBT.

## Methods

This study utilized 3 samples: a discovery sample, a validation sample and a treatment response sample.

### Discovery and Validation Samples

The discovery and validation samples were both drawn from the Twins Early Development Study (TEDS). TEDS is an ongoing longitudinal study of more than 11,000 twin pairs born in England and Wales in 1994, 1995 and 1996, which has been shown to be representative of the UK population [23]. The discovery sample included 1,026 MZ twin pairs from TEDS for whom genome-wide genotyping data were available, as well as data on emotional problems at age 12 years. The validation sample included a further 1,409 unrelated individuals from TEDS (a randomly selected individual from the remaining dizygotic twins pairs) with available data.

#### Measures

Emotional problems were measured in the discovery and validation samples at age 12 years using the emotional symptoms subscale of the Strengths and Difficulties Questionnaire [24]. We created a composite score by summing the z scores from child and parent reports and dividing by 2. Parenting was assessed at age 12 years in the validation sample using 2 child report measures: the Parental Feelings Questionnaire [25] and the Parental Strategies Questionnaire [26]. The Parental Feelings Questionnaire includes 7 statements on the child's relationship with their parent on a 3-point scale (very true, quite true or not true). The measure included 4 negative items (e.g. ‘I make my parents angry’) and 3 positive ones (e.g. ‘I feel happy about my relationship with my parents’). Positive items were reversed so that the total score reflected parental negativity. The Parental Strategies Questionnaire included 4 items in which children were asked to rate on a 3-point scale (rarely/never, sometimes or often) what their parent did if they misbehaved, including 2 positive (e.g. ‘explain or reason with me’) and 2 negative (e.g. ‘they give me a smack’) items. Positive items were reversed so that the total score reflected a more negative discipline strategy. An overall parenting score was created by summing the standardized scores from both scales. Separate positive and negative parenting scores were created by selecting the positive and negative items from each scale as reported previously in the TEDS data [27].

#### Genetic Data and Quality Control

Both the discovery and validation samples were genotyped as part of the larger TEDS sample. Full details of genotyping and quality control are provided elsewhere [28]. In brief, DNA was extracted from buccal cheek swab samples and genotyped using Affymetrix GeneChip 6.0 single-nucleotide polymorphism (SNP) genotyping arrays. Individuals were removed if they had a low call rate or excessive heterozygosity, an atypical population ancestry, relatedness or sample duplication or gender mismatches. SNPs were excluded if they had a call rate <98%, a minor allele frequency <1% or a Hardy-Weinberg p value <1 × 10^-20^. Following quality control, 679,050 SNPs remained for analysis.

### The Treatment Response Sample

The treatment response sample was drawn from the Genes for Treatment (GxT) study, a multi-site collaboration including 1,519 individuals which was designed to examine genetic and clinical predictors of response to psychological treatments in paediatric anxiety disorders. Full details of the sample are available elsewhere [29]. In brief, participants were included if they were aged 5-18 years (94% were 5-13 years old), met DSM-IV criteria for a primary diagnosis of an anxiety disorder and provided DNA. Parents provided written consent and children gave written or verbal assent. All sites administered the Anxiety Disorders Interview Schedule for the DSM-IV, Parent and Child Versions (ADIS-IV-C/P [30]), except for 2 sites where the German equivalent, i.e. the Kinder-DIPS, was used [31]. Participants were assessed before and immediately after treatment (post-treatment), with further assessments made 3, 6 or 12 months after treatment cessation where possible (follow-up). The severity of the primary anxiety disorder was measured at each time-point using the Clinician's Severity Rating (CSR) from the structured interview, which assigns a score of 0-8 (absent to very severe). A diagnosis was made when the child met the diagnostic criteria and received a CSR of 4 or more. Ten sites (n = 1,396) also assessed comorbid mood (major depression or dysthymia) or externalizing disorders (oppositional defiant disorder, conduct disorder or attention-deficit/hyperactivity disorder) at baseline using the ADIS-C/P. All assessments were completed by graduate assistants or clinical staff (mainly psychologists) trained in administration of the instruments. Sites have previously reported good inter-rater reliability for the diagnostic instruments using these samples [32,33,34]. At 8 sites (n = 1,289), parents also completed the Depression Anxiety Stress Scales [35], assessing depression, anxiety and stress symptoms experienced over the past week. For this study, the 3 subscales were summed to create an overall measure of parental psychopathology.

Of the 980 participants with available genome-wide genotyping data and at least 1 post-baseline assessment, 269 (27.5%) were treated with individual CBT, 503 (51.3%) with group-based CBT, 201 (21.2%) with brief parent-led CBT and 7 (0.7%) with guided self-help CBT. In order to limit the heterogeneity of the sample and aid the interpretation of treatment-specific effects, individuals treated with guided self-help CBT were excluded from the analysis. For the remaining 973 participants (54.9% female, mean age 9.8 years, SD 2.2), primary diagnoses included generalized anxiety disorder (n = 362; 37.2%), social anxiety disorder (n = 201; 20.7%), specific phobia (n = 106; 10.9%) and separation anxiety disorder (n = 223; 22.9%). The remaining participants (n = 81; 8.3%) were grouped as ‘other’ anxiety disorders, which included panic disorder with and without agoraphobia and agoraphobia without panic disorder (n = 26), obsessive-compulsive disorder (n = 34), post-traumatic stress disorder (n = 13), selective mutism (in patients with primary selective mutism, a diagnosis of severe social anxiety disorder was also given; the selective mutism was considered by the clinician to be primary, the most interfering, n = 2) or anxiety disorder not otherwise specified (n = 6).

#### Genetic Data and Quality Control

Genotyping and quality control procedures for the GxT study are documented elsewhere [36]. In brief, DNA was extracted from buccal swabs and saliva and genotyped on Illumina Human Core Exome-12v1.0 microarrays. Individuals with a call rate <99% or excessive heterozygosity were removed, as were those with gender mismatches or evidence for relatedness or sample duplication. SNPs were excluded if they had a call rate <99%, a minor allele frequency <5% or a Hardy-Weinberg p value lower than 1 × 10^-5^. Quality-controlled data was imputed to the December 2013 release of the 1,000 Genomes Project using IMPUTE2. Only SNPs with an information metric >0.8 and a minor allele frequency >1% were retained for analysis.

### Analyses

#### Discovery Sample

Discordance in emotional symptom scores was calculated as the absolute difference in scores between members of the pair. The effects of age, sex and the twin pair's mean score on emotional symptoms were regressed out to create a residual score, which was then included as an outcome variable in a linear regression in PLINK. In order to control for possible effects of population stratification, we included the first 10 principal components from previous analyses of the TEDS data [28] as covariates in all analyses.

#### Validation Sample

In the validation sample, we aimed to test whether the environmental sensitivity polygenic score moderated the effects of parenting on emotional problems. Polygenic scores were calculated for each individual in the sample using the β and p values from the discovery sample.

We used increasingly liberal significance thresholds to select 8 sets of SNPs from the discovery sample that reached p < 0.001, 0.01, 0.05, 0.1, 0.2, 0.3, 0.4 and 0.5. Prior to inclusion, SNPs were pruned for linkage disequilibrium (LD) using p value-informed clumping in PLINK employing cut-offs of LD (r^2^ = 0.25) and distance (a 200-kb window).

As in the discovery sample, we created a standardized age- and sex-regressed residual score of emotional symptoms for individuals in the validation sample. We explored the main effects of the polygenic environmental sensitivity score and parenting on this outcome using linear regressions. Next, we tested whether the polygenic score moderated the effects of parenting on emotional problems by testing a polygenic score × parenting interaction term in these models. The presence of a gene-environment correlation (i.e. an effect of the polygenic score on parenting) could potentially bias any polygenic score × parenting interactions. We therefore also tested whether our measures of parenting were associated with the polygenic score using linear regressions. We included socio-economic status as a covariate, as well as the first 10 principal components previously derived from genome-wide analyses of the TEDS data [28], in order to account for any population stratification effects.

#### Treatment Response Sample

In the treatment response sample, we aimed to test whether the polygenic environmental sensitivity score predicted response to psychological treatments. We defined treatment response in the GxT sample as the change in severity (CSR score) of the primary anxiety diagnosis from baseline to each time point in the study including measurements from the post-treatment and 3-, 6- and 12-month time points. In order to include all of the available outcome data simultaneously, and provide estimates in the presence of missing values, we used a linear mixed model fitted with full maximum likelihood.

We constructed a model including the fixed effects of baseline severity (CSR score of the primary diagnosis at baseline, centred at the mean) and the linear and quadratic effects of time to account for the curvilinear slope of treatment outcome. To account for correlations between repeated measures from the same subject, all models included individuals as a random effect. We also included a higher-order random effect of trial to account for between-trial differences. As in previous analyses, we covaried for clinical and demographic covariates including age, sex, primary diagnosis and treatment type by including these as fixed effects. We also included the first 10 principal components generated from previous genome-wide analyses of the GxT data to account for confounding caused by population stratification.

A polygenic environmental sensitivity score was calculated for each individual in the GxT sample using the same approach as in the validation sample and entered into the above model as a fixed effect. First, we tested the effects of the polygenic score on the overall treatment response. Next, we tested treatment-specific effects by examining the effects of the polygenic score separately in participants treated with individual CBT, group CBT or brief parent-led CBT and by testing for treatment type × polygenic score interactions.

## Results

### Discovery Analyses

In total, 1,026 MZ twin pairs (56.9% female, mean age 11.28 years, SD 0.02) with available genome-wide genotyping data and data on emotional symptoms were included in the discovery analyses. None of the included 679,050 SNPs reached genome-wide significance; nevertheless, several suggestively significant findings (p < 1 × 10^-5^) were identified and are described in table 1.

### Validation Analyses

The validation sample included 1,406 unrelated individuals with available data. The sample was significantly younger than the discovery sample (mean age 11.20 years, SD 0.70, t = 2.53, p = 0.010) and included significantly fewer females (52.1%, χ^2^ = 5.33, p = 0.021). However, individuals did not differ in their mean emotional symptoms scores (t = −1.50, p = 0.133).

All 679,050 SNPs from the discovery analysis passed quality control in the validation sample, and following LD-based pruning 155,019 SNPs remained to calculate the polygenic environmental sensitivity score. We generated 8 scores using increasingly liberal significance thresholds to select SNPs from the discovery sample (p < 0.001, 0.01, 0.05, 0.1, 0.2, 0.3, 0.4 and 0.5), which included 400; 3,161; 13,632; 25,384; 46,752; 66,205; 84,025, and 100,111 SNPs, respectively.

**Fig. 1:**
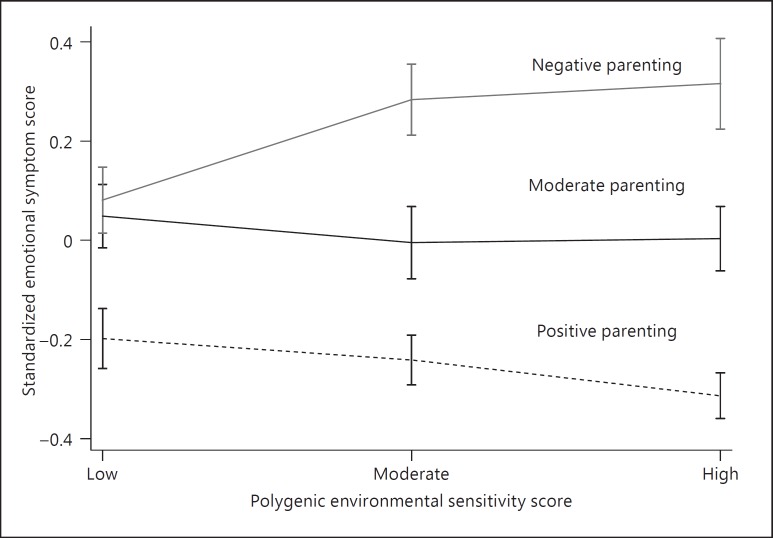
Effects of the polygenic environmental sensitivity score, parenting and their interaction on emotional problems. Mean standardized emotional symptom score by tertiles of parenting (representing negative, moderate and positive parenting) and tertiles of the polygenic environmental sensitivity score (low, moderate and high, threshold: p < 0.5). Error bars represent 1 standard error.

Table 2 shows the results of a linear regression exploring the main effects of each polygenic environmental sensitivity score and the main effects of parenting on emotional problems. The polygenic score was not significantly associated with the emotional symptom score, and findings were consistent across all significance thresholds. There was a significant main effect of parenting on emotional problems in the expected direction, with more negative parenting associated with increased emotional symptom scores. In order to investigate whether the polygenic environmental sensitivity score moderated the effects of parenting on emotional problems, we added an interaction term to the above models. Significant interactions were identified for polygenic scores calculated using 5 of the 8 p value thresholds. Interaction effects began to emerge when a threshold of p < 0.1 was used in the discovery sample where they explained an additional 0.33% of the variance. The addition of further SNPs strengthened these effects, which were greatest for the polygenic scores based on a threshold of p < 0.5 where the interaction term explained an additional 0.53% of the variance. The interaction from this model is illustrated in figure 1, in which the polygenic score (based on a threshold of p < 0.5) is divided into equal tertiles to represent low, moderate and high scores and the parenting score is separated into equal tertiles to represent negative, moderate and positive parenting. Findings were in the expected direction. Specifically, for individuals with a low polygenic environmental sensitivity score, parenting had little effect on emotional problems. However, for those with a higher polygenic score, negative parenting was associated with an increased emotional symptom score, while positive parenting was associated with decreased scores.

To explore these interaction effects further, we re-analysed the data considering the effects of positive and negative aspects of parenting separately (see online suppl. tables S1, S2; for all online supplement material, see www.karger.com/doi/10.1159/000444023). Findings were consistent with those from the above analyses. Specifically, in individuals with a high polygenic score, negative parenting was associated with increased emotional problems, while positive parenting was associated with decreased emotional symptom scores. Conversely, in those with a low polygenic score, neither positive nor negative parenting had an effect on emotional problems. There was no evidence for a gene-environment correlation. That is, there was no significant association between the polygenic environmental sensitivity score at any of the measured thresholds and any of our measures of parenting (online suppl. table S3). Finally, to test whether the same interaction effects were observed across raters, we re-analysed the data using child-reported emotional problems and parent-reported parenting. The findings were similar to those from our initial analysis, showing significant interaction effects which emerged when using a threshold of p < 0.2 (online suppl. table S4).

### Treatment Response Analyses

We used a linear mixed model to identify predictors of response (change in the severity of the primary diagnosis). Initially we explored the effects of clinical and demographic factors and the findings were similar to those reported for the full sample [29]. Specifically, individuals with social anxiety disorder or a specific phobia showed a significantly poorer response to treatment than those with generalized anxiety disorder (β = 0.43, p < 0.001, and β = 0.19, p = 0.013, respectively). However, treatment response did not differ according to any other factors including sex, age or treatment type (all p > 0.05).

In total, 277,893 SNPs from the discovery analysis were available in the treatment response sample, and following LD-based pruning 72,375 remained for calculation of the polygenic environmental sensitivity score. We generated 8 scores using increasingly liberal significance thresholds to select SNPs from the discovery sample (p < 0.001, 0.01, 0.05, 0.1, 0.2, 0.3, 0.4 and 0.5), which included 159; 1,295; 5,905; 10,988; 20,423; 29,461; 37,668, and 45,371 SNPs, respectively.

The polygenic score did not significantly predict overall response to treatment, and results were consistent across the different thresholds used to calculate the score (table 3). However, the polygenic score did have treatment-specific effects. Specifically, while the score was positively associated with response to individual CBT it was negatively associated with response to brief parent-led CBT. These effects only emerged when the polygenic score included SNPs reaching p < 0.05 in the discovery sample. At this threshold, it explained 1.55% of the variance of response to individual CBT and 4.80% of the variance of response to brief parent-led CBT. While the addition of further SNPs improved the p value of these associations for brief parent-led CBT, they did not substantially improve the variance explained.

We further explored the treatment-specific effects of the polygenic score on outcome by testing for treatment type × polygenic score interactions. These analyses showed that the polygenic score (based on SNPs reaching p < 0.05 in the discovery sample) significantly moderated the effect of each treatment type on outcome (individual vs. group CBT × polygenic score interaction: β = −0.13, 95% CI −0.24 to 0.02, p = 0.02; individual vs. brief parent-led CBT × polygenic score interaction: β = −0.30, 95% CI = −0.42 to 0.17, p = 3.1 × 10^-6^, and group CBT vs. brief parent-led CBT × polygenic score interaction: β = −0.15, 95% CI −0.26 to 0.04, p = 0.007).

**Fig. 2:**
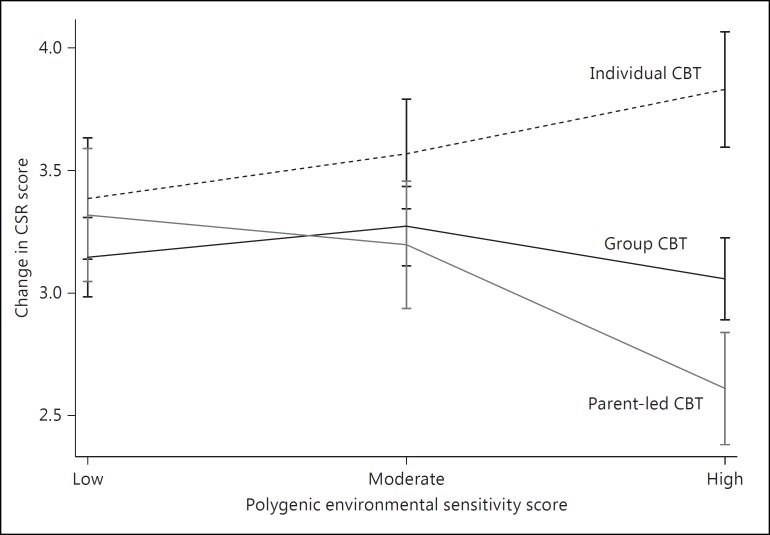
Effects of the polygenic environmental sensitivity score on the change in clinical severity rating score from baseline to the post-treatment time point. Mean change in clinical severity rating from baseline to post-treatment for individuals treated with individual CBT, group CBT and brief parent-led CBT by tertiles of the polygenic environmental sensitivity score (low, moderate and high). Error bars represent 1 standard error.

**Fig. 3:**
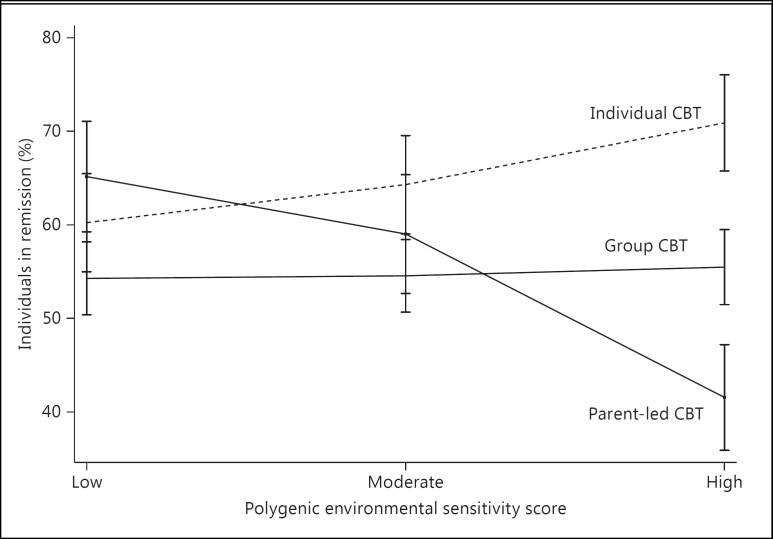
Effects of the polygenic environmental sensitivity score on the percentage of individuals in remission at the post-treatment time point. Percentage of individuals in remission at the post-treatment time point for individuals treated with individual CBT, group CBT and brief parent-led CBT by tertiles of the polygenic environmental sensitivity score (low, moderate and high). Error bars represent 1 standard error.

For those with a low polygenic environmental sensitivity score, treatment type had little effect on outcome. However, those with a high polygenic score responded well to individual CBT, moderately to group CBT and poorly to brief parent-led CBT. These effects are illustrated in figure 2, which shows the mean change in anxiety severity scores between baseline and the post-treatment time point by tertiles of low, moderate and high polygenic scores (using the threshold of p < 0.05). Figure 3 shows the percentage of individuals in remission at the post-treatment time point by tertiles of low, moderate and high polygenic scores. For those in the lower tertile, rates of remission were similar across treatment types. However, remission rates differed markedly in those with a high polygenic environmental sensitivity score (70.9%, 55.5% and 41.6%, for individual, group and brief parent-led CBT, respectively).

As all analyses included baseline anxiety severity, diagnosis, age and gender as covariates, these factors are unlikely to confound the relationship between the polygenic score and treatment response. However, we previously showed that comorbid externalizing and internalizing disorders [measured in a subset of the sample (n = 935)], as well as parental psychopathology, [measured in a smaller subset (n = 816)] were associated with treatment response. Linear and logistic regressions showed that the polygenic score was not significantly related to parental psychopathology or the presence of comorbid externalizing disorders but was significantly associated with a lower likelihood of comorbid internalizing disorders at the majority of the polygenic score thresholds tested (see online suppl. table S5).

In order to exclude the possibility that the presence of comorbid internalizing disorders confounded the relationship between the polygenic scores and treatment response, we re-ran analyses controlling for this variable on the subsample in which they were available. The findings were similar to those from the main analysis (see online suppl. table S6).

Finally, the non-random allocation of treatments meant that individuals in each treatment group differed on several clinical and demographic factors including baseline severity, diagnosis, age, parental psychopathology and comorbid externalizing and internalizing disorders (see online suppl. table S7). To ensure that interactions between polygenic score and treatment type on outcome were not biased by these differences, we used propensity score matching to restrict analyses to individuals across treatment types who were matched for baseline severity, age, diagnosis, comorbid externalizing and internalizing disorders and parental psychopathology [see online suppl. material (Methods)]. Using this reduced sample, interaction effects were of a similar magnitude to those reported for the main analyses (individual vs. brief parent-led CBT × polygenic score interaction: β = −0.28, 95% CI −0.46 to 0.09, p = 0.003, and group CBT vs. brief parent-led CBT × polygenic score interaction: β = −0.15, 95% CI = −0.29 to 0.01, p = 0.041), suggesting that they were not the result of measured differences between treatment types at baseline.

## Discussion

The differential susceptibility hypothesis suggests that the same genetic variants moderate the effects of both positive and negative environments on mental health. While several candidate gene studies support this hypothesis, this study is the first to find evidence for differential susceptibility using a genome-wide approach. We used an MZ differences design to detect variants that increase the effects of the environment on the development of emotional problems. Consistent with the differential susceptibility hypothesis, we found that a polygenic environmental sensitivity score based on these findings moderated the effects of both positive and negative parenting on emotional problems in a further sample of children. The same polygenic score also moderated response to different psychological treatments in children with anxiety disorders.

### Main Findings

We examined within-pair variability in emotional symptoms in MZ twins to detect genetic variants associated with increased sensitivity to the environment. None of our findings reached genome-wide significance. Nevertheless, suggestively significant associations were identified in a region containing *UHMK1,* a gene that encodes the brain-enriched protein kinase KIS. Animal models suggest that *UHMK1* is highly expressed in the brain, and knockdown of this gene effects the development of cortical neurons in culture [37]. In line with our findings, *UHMK1* knockout mice display a distinct deficit in fear conditioning, which is accompanied by downregulation of genes implicated in the aetiology of anxiety and fear, including multiple components of GABA_A_ receptors [38].

Consistent with our hypothesis, a polygenic environmental sensitivity score based on the whole-genome results significantly moderated the effects of parenting on emotional problems in an unrelated sample. In line with the differential susceptibility hypothesis, this interaction applied to both positive and negative aspects of parenting. In individuals with a low environmental sensitivity, parenting had little effect on emotional problems. However, in those with a high environmental sensitivity, negative parenting was associated with increased emotional problems, while positive parenting was associated with decreased emotional symptom scores. Although statistically significant, the effects of the environmental sensitivity × environment interactions were very small, explaining at most an additional 0.53% of the variance in outcome. Nevertheless, these findings are comparable to those reported for the main effects of polygenic scores and polygenic score × environment interactions in a previous study of major depression [20]. The variance explained was also larger than that reported for the main effects of polygenic scores on depression symptoms in a population sample [39].

While the polygenic environmental sensitivity score did not predict overall response to treatment, it did significantly predict differential response to individual CBT, group CBT and brief parent-led CBT. Importantly, these findings were not confounded by measured baseline clinical or demographic characteristics or biased by measured differences between treatment groups. The effects of environmental sensitivity appeared to increase linearly with the intensity of the treatment delivered, such that those with the highest environmental sensitivity responded best to individual CBT, moderately to group CBT and poorly to brief parent-led CBT. In contrast, those with a low environmental sensitivity responded equally well to each treatment type. The variance explained by the polygenic score was modest (1.62% in those treated with individual CBT and 5.77% in those treated with parent-led guided self-help) but nevertheless comparable to those in previous studies of treatment response using a polygenic approach [40].

Previous studies have created cumulative scores of environmental sensitivity based on small sets of hypothesized differential susceptibility alleles and tested them as moderators of the environment [41] and predictors of the treatment response [3]. However, this study was the first to use an MZ difference or genome-wide approach to detect and weight alleles according to their effect on environmental sensitivity. Nevertheless, our findings are consistent with those of candidate gene studies in which specific variants have been shown to enhance the effects of both negative and positive parenting on internalizing and externalizing phenotypes [14], response to CBT [13] and a range of interventions for internalizing and externalizing behaviour [15].

To our knowledge this is the first study examining the effects of sensitivity to the environment on response to CBT of varying intensity. It has been argued that individuals with a low sensitivity to the environment may require a more intensive type of treatment to achieve the same results as those who are highly sensitive. Our findings do not support this hypothesis. Outcomes for those with a low sensitivity to the environment were the same, regardless of the intensity of the treatment provided. In those with a high environmental sensitivity, the intensity of treatment was positively correlated with outcome such that they derived the most benefit from the most intensive forms of treatment. This finding is in line with the differential susceptibility hypothesis, which suggests that increasing exposure to an environment (positive or negative) has a greater effect on environmentally sensitive persons compared to environmentally insensitive individuals.

A more complete explanation may be that individuals with increased genetic sensitivity to the environment develop more of the cognitive biases underlying anxiety disorders (such as a bias towards threat) [42] and therefore require more intensive treatments to overcome these aberrant cognitions. A prospective longitudinal study with data both at the onset of illness and throughout treatment would be necessary to directly test this hypothesis. Such a design would allow for the investigation of aetiological factors [43], as well as the effects of the course of illness and disease progression [44].

### Implications

If replicated, our findings may have several important implications for understanding the aetiology of emotional problems and treatment response.

We found that our polygenic environmental sensitivity score was only a significant moderator of parenting or treatment response when it included variants reaching thresholds of p < 0.1 and p < 0.05, respectively, in the discovery dataset. This suggests that sensitivity to the environment, rather than being the result of the effects of a handful of candidate genes, is a polygenic trait, which is due to the aggregate effects of tens of thousands of variants of small effect. These polygenic effects may explain why previous studies of gene-environment interactions, which focus on a single candidate gene, often fail to replicate.

A previous polygenic score study, which assumed a diathesis stress model, suggested that around 0.5% of the variance in liability to major depression is accounted for by gene-environment interaction [20]. We report that a similar amount of variance in the aetiology of emotional problems is accounted for by variants operating in a manner consistent with differential susceptibility. A significant role of such variants may explain why, despite moderate estimates of heritability, attempts to identity the genetic variants responsible for child anxiety and depression have so far been unsuccessful [28]. They may also explain why SNP level heritability estimates are also considerably lower than those predicted from twins, even within the same samples [45].

Polygenic predictors of environmental sensitivity may allow a more accurate identification of those who are at risk of developing disorders in the face of adversity but also those who are most likely to benefit from intensive treatments. Previous findings from the current sample suggested that treatment type had little overall effect on outcome [29]. Indeed, a recent meta-analysis reported that individual CBT or group CBT was as effective as lower-intensity self-help approaches [2]. However, our results suggest that the efficacy of different treatment types differs markedly according to environmental sensitivity. These effects are potentially clinically meaningful, with remission rates in the upper tertile of the polygenic score of 70.9, 55.1 and 40.6% for individual CBT, group CBT and brief parent-led CBT, respectively. If replicated, our findings suggest that for those with a relatively low genetic sensitivity to the environment, more cost-effective, lower-intensity approaches are equally as effective as face-to-face treatment. More importantly, they also suggest that response rates may be substantially improved by targeting those with an increased genetic sensitivity to the environment with the most intensive psychological therapies.

### Strengths and Limitations

It has been noted that there are two principal challenges facing gene-environment interaction research: the necessity to develop methods which include the whole genome and the need to develop those which include and reliably measure the whole ‘environome’ of relevant environments [18]. By assessing the aggregate effects of genetic variants from across the genome on unmeasured non-shared environmental effects, the current study simultaneously addresses both of these challenges. Nevertheless, our findings should be interpreted in the light of several important limitations.

First, while our discovery analyses used a large, well-characterized sample of MZ twins, it was only adequately powered (80%) to detect individual variants with moderate effects on environmental sensitivity at genome-wide significance (explaining more than 1% of the variance of the variance) [21]. Although our polygenic approach did not rely solely on genome-wide significant findings, the discovery, validation and treatment samples were smaller than recommended for polygenic score analyses, particularly when testing treatment-specific effects [19]. Our findings should therefore be considered exploratory, pending replication in further, larger samples.

Second, we aimed to identify genetic variants that moderated the effects of the non-shared environment on emotional problems. We chose to validate these findings by exploring the interaction between our polygenic environmental sensitivity score and child-reported parenting as this is one of the most robust environmental predictors of child anxiety [46] and it has been shown to be moderated by genetic factors in a manner consistent with differential susceptibility [7]. We identified similar interactions in cross-rater analyses (using parent-rated parenting and child-rated emotional problems), and the same variants also moderated response to psychological treatment. However, it remains unknown whether these findings extend to more objectively measured environments such as observed parenting.

Finally, our treatment response sample included children with anxiety disorders receiving psychological treatment as part of a trial or treatment as usual in one of multiple studies [29]. The subsequent non-random allocations of treatments meant that treatment type was associated with several clinical and demographic characteristics at baseline. While additional analyses using propensity score matching allowed us to conclude that our findings were not biased by measured differences between treatment groups, we cannot exclude the possibility that individuals differed by unmeasured factors. Replication of our findings in a randomized trial comparing low- and high-intensity CBT is therefore necessary to fully exclude the effects of confounding by indication.

## Conclusion

The limited power provided by the cohorts used in each stage of our study means that our results should be considered exploratory until successfully replicated in larger samples. Nevertheless, if replicated, our findings suggest that responsivity to the environment is the result of multiple genetic variants of small effect rather than a few select candidate genes. We showed that these variants moderated the effects of parenting on the development of emotional problems in children. The same variants also predicted a differential response to psychological treatments, such that those with the greatest sensitivity to the environment appeared to benefit the most from more intensive types of treatment. In line with previous polygenic score studies, the gene × environment effects we identified explained a very small proportion of the variance (0.53%). However, the variance explained by gene × treatment effects was larger (1-5%). The potential clinical utility of these findings warrants further investigation of these effects in patients receiving low- and high-intensity CBT.

## Disclosure Statement

Schneider is an author of the Diagnostisches Interview bei psychischen Störungen im Kindes- und Jugendalter, from which she receives royalties. Creswell is joint author of a book used in treatment within the Overcoming trial and receives royalties from sales of the book. Rapee is an author of the Cool Kids program but receives no direct payment from it. Hudson is an author of the Cool Kids program but receives no direct payment from it. Keers, Coleman, Lester, Roberts, Breen, Thastum, Bögels, Heiervang, Meiser-Stedman, Nauta, Thirlwall, Lewis, Plomin and Eley report no potential conflicts of interest.

## Figures and Tables

**Table 1:** Associations with intra-pair differences in emotional problems in MZ twins reaching suggestive significance (p<1×10 ^5^)

Chromosome	SNP ID	Position	Allele	β	p value	Nearest gene
1	rs12131428	162426451	C	0.3885	2.10 × 10^–7^	UHMK1
22	rs5748871	17603477	A	−0.1915	1.63 × 10^–6^	CECR6
19	rs7339483	24462409	G	0.3683	6.20 × 10^–6^	ZNF254
5	rs3864261	72358254	A	0.2662	7.33 × 10^–6^	FCHO2
8	rs10875469	142333425	T	−0.2144	9.29 × 10^–6^	GPR20
5	rs1392412	72362289	G	0.2631	9.41 × 10^–6^	FCHO2

**Table 2:** Validation analyses: linear regression examining the main effects of polygenic environmental sensitivity score and parenting and their interaction on emotional problems

p value threshold	Main effects of polygenic environmental sensitivity score^a^			Main effects of parenting^a^			Polygenic environmental sensitivity score × parenting interaction^b^			
	^β^	95% CI	^p^	^β^	95% CI	^p^	^β^	95% CI	^p^	R^2^, %
0.001	−0.01	−0.04 to 0.05	0.869	0.21	0.17 to 0.25	4.43 × 10^–22^	−0.04	−0.08 to 0.01	0.107	0.05
0.01	−0.01	−0.05 to 0.03	0.718	0.21	0.17 to 0.25	4.65 × 10^–22^	0.01	−0.05 to 0.04	0.848	0.01
0.05	0.01	−0.03 to 0.05	0.615	0.21	0.17 to 0.25	4.54 × 10^–22^	0.04	−0.01 to 0.08	0.085	0.22
0.1	0.02	−0.03 to 0.06	0.470	0.21	0.17 to 0.25	4.38 × 10^–22^	0.05	0.00 to 0.09	0.035	0.33
0.2	0.01	−0.03 to 0.05	0.727	0.21	0.17 to 0.25	4.58 × 10^–22^	0.06	0.01 to 0.10	0.011	0.47
0.3	−0.01	−0.05 to 0.04	0.787	0.21	0.17 to 0.25	4.60 × 10^–22^	0.06	0.01 to 0.10	0.012	0.46
0.4	−0.01	−0.05 to 0.03	0.636	0.21	0.17 to 0.25	4.52 × 10^–22^	0.06	0.02 to 0.10	0.008	0.49
0.5	−0.01	−0.05 to 0.03	0.640	0.21	0.17 to 0.25	4.49 × 10^–22^	0.06	0.02 to 0.10	0.005	0.53

a Models included the main effects of polygenic environmental sensitivity score and child-reported parenting on age- and sex-regressed combined child-/adult-rated emotional symptom scores.

b Models included the main effects of polygenic environmental sensitivity score and child-reported parenting and their interaction on age- and sex-regressed combined child-/adult-rated emotional symptom scores. To account for possible effects of population stratification, all models also included the first 10 principal components previously derived from genome-wide analyses of the TEDS data.

**Table 3:** Treatment response analyses: linear mixed model examining the effect of the polygenic environmental sensitivity score on treatment response (change in the severity of the primary anxiety disorder)

p value threshold	Overall response				Response to individual CBT				Response to group-based CBT				Response to brief parent-led CBT			
	ß	95% CI	p	R^2^, %	ß	95% CI	p	R^2^, %	ß	95% CI	p	R^2^, %	ß	95% CI	p	R^2^, %
0.001	0.01	−0.03 to 0.05	0.699	0.03	−0.01	−0.09 to 0.08	0.865	0.00	0.02	−0.04 to 0.08	0.482	0.08	0.04	−0.06 to 0.15	0.408	0.45
0.01	0.02	−0.02 to 0.06	0.357	0.04	−0.08	−0.17 to 0.01	0.077	0.72	0.04	−0.02 to 0.10	0.182	0.23	0.07	−0.02 to 0.16	0.151	0.80
0.05	0.03	−0.02 to 0.07	0.267	0.05	−0.12	−0.21 to −0.03	0.009	1.62	0.02	−0.04 to 0.08	0.456	0.10	0.18	0.09 to 0.27	6.97 × 10^–5^	4.80
0.1	0.02	−0.02 to 0.07	0.339	0.03	−0.11	−0.19 to −0.02	0.014	1.50	0.01	−0.05 to 0.07	0.720	0.04	0.20	0.11 to 0.29	1.92 × 10^–5^	5.21
0.2	0.02	−0.02 to 0.07	0.277	0.05	−0.09	−0.18 to −0.01	0.033	1.11	0.01	−0.05 to 0.07	0.841	0.03	0.21	0.12 to 0.30	6.14 × 10^–5^	5.77
0.3	0.02	−0.03 to 0.06	0.420	0.02	−0.10	−0.18 to −0.01	0.022	1.23	0.01	−0.06 to 0.06	0.947	0.00	0.20	0.11 to 0.29	1.99 × 10^–5^	5.20
0.4	0.02	−0.03 to 0.06	0.485	0.01	−0.10	−0.19 to −0.02	0.017	1.36	0.01	−0.06 to 0.06	0.971	0.01	0.19	0.10 to 0.28	5.78 × 10^–5^	4.81
0.5	0.02	−0.03 to 0.06	0.471	0.01	−0.11	−0.19 to −0.02	0.014	1.44	0.01	−0.06 to 0.06	0.918	0.00	0.19	0.10 to 0.29	3.47 × 10^–5^	5.14

To account for data collected longitudinally, all models included the random effects of participant and the linear and quadratic effects of time. All models also included sex, age (centred), primary diagnosis (generalized anxiety disorder, social anxiety disorder, specific phobia, separation anxiety disorder or Other anxiety disorder) and treatment type (individual-based CBT, groupbased CBT or brief parent-led CBT). All models included the random effects of trial. Regression weights (ß) significantly greater than zero indicate that this variable is associated with a poorer response following treatment.
